# Segmental pigmentation disorder: A rare form of cutaneous dyspigmentation

**DOI:** 10.22088/cjim.8.3.223

**Published:** 2017

**Authors:** Kamran Balighi, Kambiz Kamyab, Arghavan Azizpour, Elahe Amini, Shabnam Fahim

**Affiliations:** 1 Depatment of Dermatology, Razi Hospital, Tehran University of Medical Sciences, Tehran, Iran.; 2 Razi Hospital, Tehran University of Medical Sciences, Tehran, Iran.

**Keywords:** Segmental pigmentation disorder, Checkerboard pattern, Dyspigmentation

## Abstract

**Background:**

Segmental pigmentation disorder (SegPD) is a rare type of cutaneous dyspigmentation. This hereditary disorder, first described some 20 years ago, is characterized by hypo and hyperpigmented patches on the trunk, extremities and less likely on the face and neck. These lesions are considered as a type of checkerboard pattern.

**Case Presentation:**

Herein, we present a 26-year-old male who presented with hyperpigmented patches on his trunk, neck and upper extremities. Considering the clinical and histopathological findings, the diagnosis of SegPD was confirmed.

**Conclusion:**

SegPD is a somewhat neglected entity which should be considered in differential diagnosis of pigmentation disorders.

**I**n 1983, Metzker et al. first introduced the term segmental pigmentation disorder for pigmentation disorders with somewhat dermatomal distribution. Later in 1993, Happle et al described classic patterns of cutaneous lesions due to mosaicism. These patterns include blaschkoid, checkerboard and phylloid pattern. Blaschkoid lesions follow Baschko lines of the skin. Phylloid pattern is manifested by multiple leaf like patches mostly on the trunk and with no obvious midline demarcation (1, 2). Checkerboard pattern is characterized by block like cutaneous lesions on the trunk, extremities and less commonly on the face and neck. These lesions have a sharp midline demarcation along with less defined lateral borders. Segmental pigmentation disorder (SegPD) is characterized by hyperpigmented lesions with a checkerboard pattern which most commonly present at birth or first few weeks of life and with no common associated systemic symptoms (1). Herein, we present a case of SegPD to revive this entity in our population in Iran.

## Case Presentation

**Figure 1:**
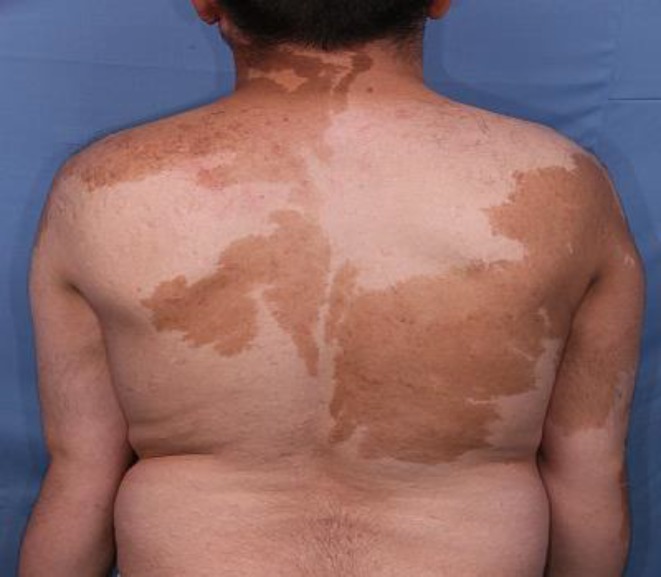
Squares of hyperpigmentation over the anterior aspect of the trunk and upper extremities with midline demarcation on the trunk.

**Figure 2:**
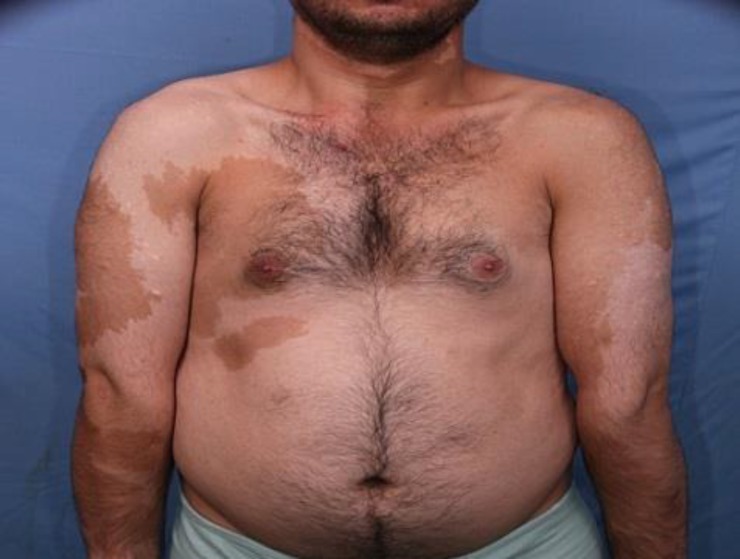
Segmental and block-like hypo-or hyperpigmentation patches with sharp midline demarcation on the dorsal aspect of the trunk.

A 26-year-old male presented to our outpatient department in Razi Hospital, Tehran, Iran affiliated to Tehran University of Medical Sciences with complaint of patchy discoloration on his trunk and upper limbs. These lesions had developed soon after birth and enlarged proportional to his growth and stabilized during adolescence. He had been treated with 4 sessions of Q-switch laser which was unsuccessful. On physical examination, few patches of hyperpigmentation were noted on the trunk, neck and upper extremities (figure 1). These lesions demonstrated a sharp midline demarcation particularly on the back (figure 2).

No other remarkable physical signs were found on physical examination. He was not taking any regular medications and was otherwise well with no systemic symptoms. Family history for similar lesions was negative. A punch biopsy of hyperpigmented lesions revealed basal layer pigmentation of the epidermis along with mild perivascular lymphocytic infiltration in the dermis (figure 3a) which was confirmed by Fontana stain of the sample (figure 3b). 

**Figure 3a:**
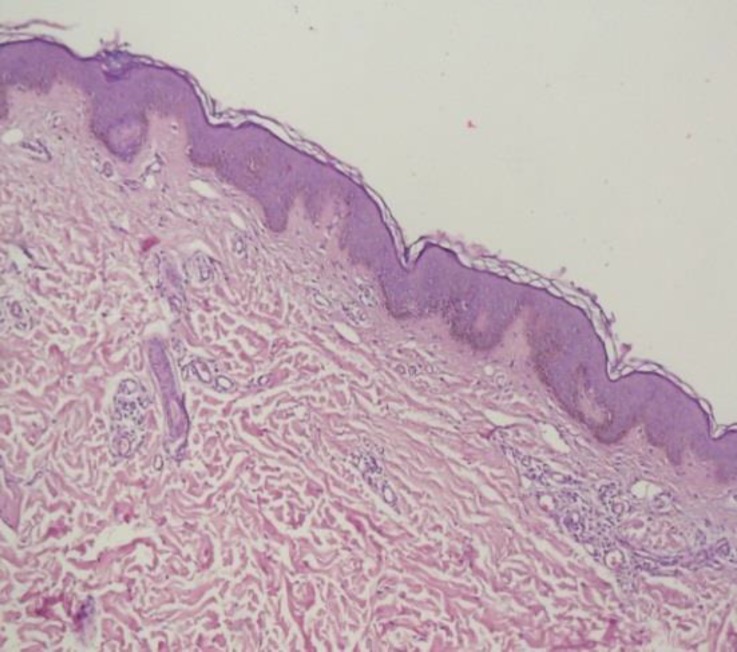
Hematoxylin and eosin staining of hyperpigmanted lesion demonstrating basal pigmentation (x100)

Melanocytes were not increased in number and no nevic cells were observed. Biopsy specimen from uninvolved skin was insignificant. Considering these findings, the diagnosis of segmental pigmentation disorder was confirmed.

**Figure 3b:**
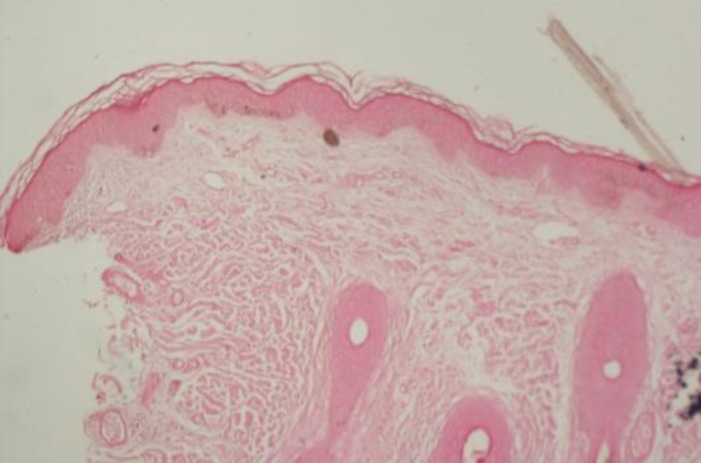
Fontana stain demonstrating alternative focal hyperpigmentation(x40

## Discussion

Segmental pigmentation disorder (SegPD) is a type of pigmentary mosaicisim disorder first described by Metzker et al. in 1983 and revised by Hogeling and Friedan in 2010. Although rare case reports of this type of dyspigmentation had appeared in the literature before, Metzker et al were first to describe and label this entity as a distinct disorder (3).

 SegPD is characterized by a block like or checkerboard pattern of hypopigmentation, hyperpigmentation, or both (4, 5). Lesions usually become apparent during childhood and early adolescence, suggesting a developmental origin (5). Incidence rate for SegPD has been estimated to be 0.35 percent per year which makes it a rather common but usually a neglected disorder (5-8). However, it was previously believed that SegPD spontaneously fade away over time, many new classic cases of SegPD which did not fade away, point towards a dual nature for this disease (2). The “checkerboard” pattern of mosaic which is consistent with the pattern of pigment alterations in our case is characterized by alternate areas of pigmentary disturbance in each hemibody, with a sharp midline demarcation. These patches of hypo- or hyperpigmentation are often located on the torso followed by the extremities, face and neck. SegPD lesions show a sharp midline demarcation especially on the ventral side of trunk rather than the dorsum, but in our case, the midline demarcation was more pronounced on the back. Lateral borders of these patches are less defined as seen in our patient. This disorder remains stable throughout life and usually has no or extremely rare associated extracutaneous anomalies including nervous system diseases (5, 9). The histopathological findings depend upon whether hyper- or hypopigmentation are evaluated. The hyperpigmented patches usually reveal only basal hyperpigmentation; suggesting increased melanin production with no increase in melanocytes. This type of basal pigmentation in histopathology of the lesions can be confused with classic café-au-lait macules (4) Therefore, the current criteria for the diagnosis of SegPD are mainly clinical (5, 10, 11).

Histopathological findings of hypopigmented patches reveal slight decrease in melanin content along with normal or decreased number of melanocytes (5) which may lead to misdiagnosis if not interpreted correctly. An important differential diagnosis, which can be easily confused with SPD, is the giant or segmental café-au-lait macules (CALMs). They do not demonstrate sharp midline demarcation and usually manifest as round or oval macules ranging in color from dark to light brown (“coffee with milk”) with smooth or irregular borders and without fading at their lateral border, but may share the same histology as SPD with melanin macroglobules which are occasionally present in café-au-lait macules (4-9). Segmental neurofibromatosis (type V) is also considered as a differential diagnosis of SegPD. Additional features of the disease, such as neurofibromas, axillary freckling and smaller sports and more numerous CALMs are in favor of segmental neurofibromatosis (12). 

Orion et al. suggest that the catch-all term SegPD be changed to segmental pigmentation anomaly (SPA) which can be used as a family name for an array of pigmentation disorders and the term SPD changed into segmental pigmentation disorder simplex" (Metzker type) and considered as a type of SPA. Segmental café-au-lait is another entity and a type of SPA in this classification (4). These criteria along with the pattern of distribution of cutaneous lesions were used in making the right diagnosis in our patient. Our patient was ultimately diagnosed with SegPD. SegPD is not treatable and has no known pattern of inheritance. To recognize the underlying specific genetic mutation(s) is the exact description and clasiification of this disease. Since reports of this disorder are extremely rare in our population, recognition of clinical and histological characteristics of this disorder may help us in early diagnosis and estimation of incidence of SegPD in our population.

## Conflict of Interest:

None declared.
